# Construction Notes

**Published:** 1893-03-18

**Authors:** 


### CONSTRUCTION NOTES.

The Committee of the Wrexham Infirmary have a proposal under consideration to provide additional accommodation for the nurses, the cost of which alteration is estimated at about £300, which amount will have to be raised by subscription.

Temporary provision for the admission of additional patients to the Hunstanton Convalescent Home has been secured by the hiring of a neighbouring house—which is estimated to afford accommodation for twenty additional patients. The committee of the Hunstanton Home report favourably upon the improvements afforded by the overhauling of the drainage and sanitary arrangements earlier in the year.

The Rural Sanitary Authority of Blaby have decided on the erection of a temporary small-pox hospital. The architect, Mr. Simpson, has been instructed to prepare plans for the same to contain two male and two female wards; the total expenditure not to exceed £100. The hospital is planned to be of corrugated iron, as in the event of a joint hospital being established this building would be useful. A fully equipped ambulance is to be purchased for the conveyance of the sick.

The extension of the Luton Cottage Hospital has been fully carried out by the lengthening of the wards, accommodation being now ready for 27 patients. The total cost of this improvement was £650, of which sum £400 has already been paid to the contractors, Messrs. Neville Brothers. The committee call attention to the fact that the enlargement of the institution will necessitate increased expenditure for its maintenance, which, it is estimated, will not be less than £800 during the current year.

The local authorities of Perth have contributed £1,500 to the Perth City and County Hospital, towards the erection of new washing houses, laundries, with other necessary apparatus in connection with the infectious diseases wards of the hospital. As a necessary site for this extension, the directors have purchased two tenements at a cost of £1,831 18s., the ground being on the eastern side of the infirmary property. The building and engineering works are estimated to cost £2,350, and the work will be completed in a month's time.

The Cumberland Infirmary, having raised the stipulated sum of £2,000 towards the erection of the Nurses' Home, the Committee have appointed Mr. Charles Ferguson their architect, and a committee of ladies and gentlemen to consider the question of accommodation, site, &c. In September last the Building Committee presented their report, and revised tenders amounting to £2,190 8s. were accepted, and the building operations will be begun on March 1st. The amount received by the treasurer is £2,285. A sum of £700 at the least is still required.

Extensive alterations and improvements have taken place in the Turner Memorial Hospital at Keith, through the generosity of Mr. Kynoch, of Isla Bank. The new addition to the hospital is connected with the present building by a spacious corridor 40 feet long. This leads to the fever wards, two in number, each measuring 20 by 21 feet, for the accommodation of four patients. A ward for female patients is at the south end. A lavatory and other accommodation has been added to each ward, and everything relating to the sanitation of the wards has been planned on the latest and most approved principles. There is a nurses' room and two bath rooms. The entire length of this new addition to the hospital measures about 90 feet. The architect is Mr. F. D. Robertson.

The Durham Urban Sanitary Authority have finally decided to erect an Infectious Diseases Hospital at the cost of £660, upon a site which the Dean and Chapter have generously granted for the purpose at a nominal rate for a long lease. The new hospital will comprise two wings, with 12 beds in each. Messrs. Whaley and Coates are the builders.

Plans from Mr. Simpson for the proposed Infectious Diseases Hospital at Leith have met with the full approval of the committee of management. These plans show five wards, one of which occupying a central position is a probationary ward, placed at a distance of 80 feet from the other wards. Each of the wards is to contain eighteen beds, the entire number of beds in the hospital numbering eighty-two. The wards will be heated, partly by open fire-places, partly by low-pressure hot-water pipes. The disinfecting house is to be isolated from the central building. The administration block is to provide for a medical officer, nurses, maids, and general servants. The total cost of the hospital is estimated at £24,693 0s. 11d.

